# Zika vaccines and therapeutics: landscape analysis and challenges ahead

**DOI:** 10.1186/s12916-018-1067-x

**Published:** 2018-06-06

**Authors:** Annelies Wilder-Smith, Kirsten Vannice, Anna Durbin, Joachim Hombach, Stephen J. Thomas, Irani Thevarjan, Cameron P. Simmons

**Affiliations:** 1 Immunization, Vaccines & Biologicals, World Health Organization, Geneva, Switzerland; 2 Lee Kong Chian School of Medicine, Nanyang Technological University, Singapore, Singapore; 3 Department of Epidemiology and Global Health, Umea University, Umea, Sweden; 4 Center for Immunization Research, Johns Hopkins Bloomberg School of Public Health, Baltimore, MD USA; 5 State University of New York, Upstate Medical University, Syracuse, NY USA; 6 Doherty Institute for Infection and Immunity, Parkville, VIC 3010 Australia; 7 The Royal Melbourne Hospital, Parkville, VIC 3010 Australia; 8 Oxford University Clinical Research Unit, 764 Vo Van Kiet street, District 5, Ho Chi Minh City, Vietnam; 9 Institute of Vector-borne Disease, Monash University, Melbourne, VIC Australia

**Keywords:** Zika, Zika vaccines, Flavivirus, Anti-virals, Prophylaxis, Therapeutics, Efficacy trials, Zika diagnostics, Monoclonal antibodies, Immune correlates, Immune surrogates, Human controlled infections, Clinical endpoints

## Abstract

**Background:**

Various Zika virus (ZIKV) vaccine candidates are currently in development. Nevertheless, unique challenges in clinical development and regulatory pathways may hinder the licensure of high-quality, safe, and effective ZIKV vaccines.

**Discussion:**

Implementing phase 3 efficacy trials will be difficult given the challenges of the spatio-temporal heterogeneity of ZIKV transmission, the unpredictability of ZIKV epidemics, the broad spectrum of clinical manifestations making a single definite endpoint difficult, a lack of sensitive and specific diagnostic assays, and the need for inclusion of vulnerable target populations. In addition to a vaccine, drugs for primary prophylaxis, post-exposure prophylaxis, or treatment should also be developed to prevent or mitigate the severity of congenital Zika syndrome.

**Conclusion:**

Establishing the feasibility of immune correlates and/or surrogates are a priority. Given the challenges in conducting phase 3 trials at a time of waning incidence, human challenge trials should be considered to evaluate efficacy. Continued financial support and engagement of industry partners will be essential to the successful development, licensure, and accessibility of Zika vaccines or therapeutics.

## Background

The devastating consequences of Zika virus (ZIKV) infection, leading to congenital Zika syndrome (CZS) and neurological complications such as Guillain–Barre Syndrome (GBS), led the World Health Organization (WHO) to declare a Public Health Emergency of International Concern on February 1, 2016 [1], and to call on the global research and product development (R&D) communities to prioritize the development of preventative and therapeutic solutions [2]. The R&D communities responded rapidly, with 45 vaccine candidates being initially evaluated in non-clinical studies and most progressing to active development. Of these, several have advanced beyond pre-clinical studies in animals and entered phase 1 human trials [3, 4], with two candidates having entered phase 2 trials (https://clinicaltrials.gov/ct2/show/NCT03110770, https://clinicaltrials.gov/ct2/show/NCT03014089). Additionally, the role of therapeutic and prophylactic medicinal products in the management of ZIKV infections in pregnant women and other high-risk groups remains to be determined. Herein, we describe the various vaccine platforms, with a discussion on their advantages and disadvantages in the context of use scenarios, and provide an overview of the current status of vaccine development. Furthermore, we propose three plausible clinical indications for prophylactic or therapeutic agents against ZIKV. Both vaccines and therapeutics must be evaluated for their efficacy in human trials, yet the design of efficacy trials and the appropriate selection of clinical endpoints pose a challenge. In particular, the rapid decline in Zika cases in the second year following the Public Health Emergency of International Concern declaration has put clinical efficacy trial feasibility at stake. We discuss options on how best to address these hurdles.

## ZIKV vaccines

WHO has outlined two use scenarios for a ZIKV vaccine [5], namely in emergency outbreak response and for endemic transmission. Emergency outbreak response involves a targeted mass vaccination during an ongoing epidemic or an imminent outbreak of ZIKV to prevent ZIKV-associated disease in women of child-bearing age in order to mitigate CZS. Endemic transmission use involves a broad or universal vaccination campaign of the general population in the inter-epidemic period, extending from early childhood to adults, followed by routine immunization, in order to establish population immunity to prevent transmission, and ultimately to prevent ZIKV-related adverse birth outcomes and neurological complications.

Based on current knowledge on the transmission of ZIKV and experiences with past disease outbreaks, WHO has prioritized the development of vaccines suitable for use in an emergency or outbreak scenario. Therefore, and in line with the WHO Zika Strategic Response plan, WHO developed a Target Product Profile for a ZIKV vaccine for emergency use where immunization of women of reproductive age is considered to be of highest priority [5]. Although WHO declared an end to its global health emergency over the spread of ZIKV on November 18, 2016, the long-term need for a ZIKV vaccine continues [6]. Under the Blueprint Plan of Action [7].

WHO led a series of initiatives to maintain continuous dialogue between developers, regulators, and public health experts to identify how best to achieve rapid, robust, safe, and evidence-based licensing of ZIKV vaccines. In June 2016, WHO hosted an expert consultation on regulatory considerations for ZIKV vaccine development, outlining vaccine platform focal points for developers and regulators, as well as the mechanisms of approval [8]. In June 2017, additional information was provided regarding clinical trial endpoints and trial site selection. WHO has also hosted periodic meetings to review the progress of ZIKV vaccine development and foster opportunities for data sharing [9, 10].

### Factors that render the development of a ZIKV vaccine feasible

Although ZIKV strains are categorized into two genetic lineages, African and Asian/American, ZIKV has been classified as a single serotype with limited strain variability [11]. Recent studies on macaques showed that immune responses primed by infection with East African ZIKV completely protected macaques from detectable viremia when subsequently re-challenged with heterologous Asian ZIKV [12]; thus, a ZIKV vaccine based on a single ZIKV strain may be sufficient. Successful vaccines have been developed for other single serotype flaviviruses such as yellow fever, Japanese encephalitis (JEV), and tick-borne encephalitis (TBEV), with well-defined correlates of protection, thus rendering the development of a monovalent vaccine against ZIKV with a favorable probability of technical and regulatory success [8]. Early findings from animal studies suggest a protective threshold of ZIKV vaccine-induced neutralizing activity that prevents viremia after acute infection, as determined after challenge with an infective dose [13, 14]. Three different vaccine platforms have been tested in non-human primate models, with all showing 100% protection against viremia following a ZIKV challenge [15, 16]. Additionally, various vaccine platforms have been tested for their ability to protect against ZIKV transmission to the fetus [17], with the findings showing markedly diminished levels of viral ZIKV RNA in maternal, placental, and fetal tissues, which resulted in protection against placental damage and fetal demise [17]. These studies are therefore a proof-of-concept that protection against CZS is possible.

### Potential hurdles to ZIKV vaccine development

Several important hurdles may impede ZIKV vaccine development. Firstly, given the early stages of development of animal models for ZIKV infection, disease, maternal–fetal transmission, and fetal infection, their relevance to the human experience requires additional validation. Current evidence suggests that even asymptomatic infections with presumably low levels of viremia in the mother could result in CZS [18]. It is unknown whether sterilizing immunity and robust T cell response are required to avert transplacental transmission of ZIKV during pregnancy [19]. Answering these questions will be critical for the development of a vaccine that protects against CZS. If sterilizing immunity is indeed required, this would set a high bar for a ZIKV vaccine since, similar to other flavivirus vaccines (e.g., JEV, dengue viruses (DENV), and TBEV), sterilizing immunity has not yet been achieved. Optimally, the efficacy afforded by a ZIKV vaccine would be durable, as protection throughout the reproductive years is desired.

Secondly, concerns have been raised about the hypothetical risk of vaccine-associated GBS given the association of natural ZIKV infection with a higher risk of GBS [20, 21]. If the mechanism of ZIKV-associated GBS is direct neuroinvasion, there could be implications for the design of neurovirulence testing of live attenuated ZIKV vaccines [8]. Conversely, if GBS is immune mediated, there could be implications for all ZIKV vaccines.

The sequence and antigenic similarity between ZIKV and DENV [22], and potentially also other flaviviruses, has led some to speculate whether pre-existing immunity to one or more flaviviruses could impact clinical outcomes following a subsequent ZIKV infection, as many of these flaviviruses co-circulate [23, 24]. Whilst in vitro studies have generated evidence in support of immune enhancement [23] between DENV and ZIKV, an increasing body of evidence from in vivo non-human primate studies [25, 26] and observational studies in humans [27] have shown a lack of association between more severe ZIKV disease and prior DENV infections, which is reassuring for vaccine development. Nevertheless, careful monitoring will be needed, and clinical trial study designs should ideally include evaluation of safety and immunogenicity, as well as of the potential for clinical benefit in both flavivirus-primed and naive populations.

### Current ZIKV vaccine platforms

Both traditional (purified inactivated, live attenuated, recombinant sub-unit) and more novel (DNA, self-replicating RNA, messenger RNA (mRNA), viral-vectored) ZIKV vaccine platforms are in development. In July 2016, WHO developed a catalog of preclinical and clinical ZIKV vaccines by searching the WHO International Clinical Trial Registry Platform [28] and the National Institutes of Health (NIH) clinical trial registry (ClinicalTrials.gov), by literature review, and by contacting research groups in academia and industry. Table 1 highlights the ZIKV vaccine candidates in clinical development as of October 2017, and Table 2 outlines ZIKV vaccine candidates in the preclinical phase as of January 2017. Additionally, WHO maintains an updated list of ZIKV vaccine clinical trials through the WHO clinical trials tracker [29]. Below, we discuss the potential advantages and disadvantages of the various platforms, and highlight selected vaccines that have entered clinical trials.

**Table 1:** WHO Zika virus vaccine pipeline: in human trials (last updated September 2017 [29])

Platform	Candidate vaccine	Immunogen	Adjuvant type	Replicating virus	Registry ID	Trial status	Sponsor name	Sponsor type	Phase	Study start date	Age	Sample size	Location
DNA	GLS-5700	prM/E	None	No	NCT02809443	Open, not recruiting	GeneOne Life Science, Inc./Inovio Pharmaceuticals	Industry	Phase 1	1/7/16	Adult	40	United States of America, Canada
					NCT02887482	Open, recruiting	GeneOne Life Science, Inc./Inovio Pharmaceuticals	Industry	Phase 1	1/8/16	Adult	160	Puerto Rico
Peptide	AGS-v	Mosquito salivary proteins		No	NCT03055000	Open, recruiting	NIH	Government	Phase 1	9/2/17	Adult	60	United States of America
Recombinant viral vector	MV-Zika	prM/E	None	Yes	NCT02996890	Open, recruiting	Themis Bioscience	Industry	Phase 1	4/4/17	Adult	48	Austria
mRNA	mRNA-1325	prM/E	None	No	NCT03014089	Open, recruiting	Moderna Therapeutics	Industry	Phase 2	1/12/16	Adult	90	United States of America
DNA	VRC-ZKADNA085–00-VP or VRC-ZKADNA090–00-VP	prM/E	None	No	NCT02840487	Open, not recruiting	NIAID	Government	Phase 1	11/7/16	Adult	120	United States of America
					NCT02996461	Open, recruiting	NIAID	Government	Phase 1	8/12/16	Adult	50	United States of America
					NCT03110770	Open, recruiting	NIAID	Government	Phase 2	29/3/17	Child, Adult	2500	United States of America, Puerto Rico
Inactivated whole target organism	ZIKV PIV	Full genome	Aluminum	No	NCT02963909	Open, recruiting	NIAID	Government	Phase 1	1/11/16	Adult	75	United States of America
					NCT02952833	Open, recruiting	NIAID	Government	Phase 1	14/10/16	Adult	90	United States of America
					NCT02937233	Open, recruiting	BIDMC	Academic	Phase 1	1/10/16	Adult	48	United States of America
					NCT03008122	Open, recruiting	NIAID	Government	Phase 1	24/2/17	Adult	90	Puerto Rico
Inactivated whole target organism	BBV121	Full genome	Aluminum	No	CTRI/2017/05/008539	Open, recruiting	Bharat Biotech International Ltd., India	Industry	Phase 1	1/6/17	Adult	48	India

*BIDMC* Beth Israel Deaconess Medical Center, *NIAID* National Institute of Allergy and Infectious Diseases, *NIH* National Institutes of Health, *PIV* purified, inactivated whole virus vaccines, *ZIKV* Zika virus

^#^Reference: http://www.who.int/immunization/research/vaccine_pipeline_tracker_spreadsheet/en/

**Table 2:** WHO – Pipeline Zika virus (ZIKV) vaccines (in preclinical development) (last updated January 2017) January 2017)

Platform	Candidate vaccine name	Developer/Collaborators	Replicating virus (Yes/No)	Antigen	Adjuvant
Inactivated whole target organism	BK1603	BIKEN	No	ZIKV full genome	To be determined
Inactivated whole target organism		Bio-Manguinhos in house development and Sanofi Pasteur/WRAIR (discontinued in 2017)	No	ZIKV full genome	Alum
Recombinant subunit (non- VLP)		Bio-Manguinhos partnership	No	ZIKV E protein	Alum
Recombinant viral vector		Bio-Manguinhos/Aggeu Magalhaes (FIOCRUZ)	Yes	PrM/E and PrM/E/NS1	None
Live, attenuated recombinant virus		Brazilian Ministry of Health agreement with University of Texas	Yes	rZIKV	None
				rZIKV NS1	
Inactivated whole target organism	Butantan ZIKV	Butantan	No	ZIKV full genome	Alum
Live, attenuated target organism	Butantan attenuated ZIKV	Butantan		ZIKV full genome	None
Inactivated virus + aluminum adjuvant	ZIKV	Emergent BioSolutions	No	ZIKV full genome	Aluminum
Recombinant viral vector	GEO-ZM05	GeoVax/University of Georgia/CDC Atlanta, US	No	ZIKV PrM/E + NS1	None
SAM	WT	GSK-NIH	Yes	ZIKV prM/E	
SAM	CO	GSK-NIH	Yes	ZIKV prM/E	
SAM	VRC_5283	GSK-NIH	Yes	ZIKV prM/E	
SAM	VRC_5288	GSK-NIH	Yes	ZIKV prM/E	
Recombinant subunit (non-VLP)	ZIK-80E	Hawaii Biotech, Inc.	No		
Recombinant subunit VLP (non-fusion)	ZIKVLP	Institut Pasteur Shanghai, China	No		
Recombinant viral vector	NI.LV-ZIK	Institut Pasteur, Paris, France	No	ZIKV prM/E	None
Recombinant viral vector	ChAdOx1-Zk	Jenner Institute		ZIKV prM/E	None
Inactivated whole target organism	KAKETSUKEN ZIKV	Kaketsuken	No	ZIKV full genome	TBD
Inactivated whole target organism		NewLink Genetics	No	ZIKV prM/E + NS1	TBD
Recombinant subunit VLP (fusion)		NewLink Genetics	No	ZIKV prM-E	TBD
Live, attenuated target organism	rZIKV/D2D30	NIAID	Yes	ZIKV prM/E	None
Live, attenuated target organism	rZIKV/D4D30	NIAID	Yes	ZIKV prM/E	None
Live, attenuated target organism	rZIKVD30	NIAID	Yes	ZIKV full genome	None
Recombinant viral vector		NIAID	Yes		None
Sf9 cells/Baculo	ZIKV envelope dimers (EnvD)	Novavax, Inc.	No	ZIKV E protein	Matrix M adjuvant or aluminum hydroxide
nanoDNA/ZIKA-LAMP chimera construct	LAMP-ZIKA nanoDNA	Pharos Biologicals	No	ZIKV prM/E	
Recombinant subunit (non-VLP)	ZIKA recombinant	Protein Sciences/Sinergium Biotech/Lab Liomont	No	ZIKV E protein	Aluminum based
Peptide	Replikins Zika Vaccine and Bocker	Replikins Ltd.	No	Synthetic Peptides	None
Recombinant viral vector	Chimerivax-Zika	Sanofi Pasteur		ZIKV prM/E + NS1	None
Recombinant viral vector	SCV-CHIKV+ZIKV+YF	Sementis Ltd.	No	ZIKV, yellow fever, and CHIKV surface antigens	None
Recombinant viral vector	SCV-CHIKV+ZIKV	Sementis Ltd.	No	ZIKV and CHIKV surface antigens	None
Recombinant viral vector	SCV-ZIKV	Sementis Ltd.	No	ZIKV surface antigens	None
Inactivated whole target organism		Takeda	No	ZIKV full genome	Alum
Subunit		Tours University	No		
VLP HBV-Zika					
Prime-boost		U1187 INSERM (CYROI, La Reunion)			
Live attenuated + ZIKV exosome					
Inactivated whole target organism		Valneva	No	ZIKV full genome	Aluminum hydroxide
Recombinant viral vector	VXA-Zikavax	Vaxart		ZIKV prM/E	
VLP	VBI-2501A	VBI Vaccines	No	ZIKV E + NS1	
DNA		WRAIR/BIDMC/Harvard	No	ZIKV prM/E	
Recombinant viral vector		WRAIR/BIDMC/Harvard		ZIKV prM/E	

*BIDMC* Beth Israel Deaconess Medical Center, *BIKEN* Research Foundation for Microbial Diseases of Osaka University, *CDC* Centers for Disease Control and Prevention, *CHIKV* Chikungunya virus, *GSK* GlaxoSmithKline, *LAMP* loop-mediated isothermal amplification, *NIH* National Institutes of Health, *NIAID* National Institute of Allergy and Infectious Diseases, *SAM* self-amplifying mRNA, *SCV* Sementis Copenhagen vector, *VLP* virus-like particles, *WRAIR* Walter Reed Army Institute of Research, *YF* Yellow fever, *ZIKV* Zika virus

#### Nucleic acid vaccines

Nucleic acid vaccines have advanced the furthest in clinical development. Both DNA plasmid-based vaccines and mRNA vaccines have utility due to their ease of production since encoding genes can easily be replaced [30], and thus have potential for scalability during an outbreak. They exhibit characteristics of subunit vaccines and live attenuated vectors, with conceptual safety advantages [22]. However, to date, neither a DNA nor an mRNA vaccine candidate has been evaluated in a phase 3 trial nor licensed for use in the prevention of another flavivirus infection, unlike live, vectored, and inactivated vaccine platforms. A limitation of DNA plasmid vaccines is the delivery technology needed for optimal protein production. For example, electroporation, i.e., the use of a pulsed electric field to introduce the DNA sequence into cells [30], would make large scale deployment in low-resource settings more difficult. A potential concern with DNA vaccines is that there might be a small possibility of chromosomal integration by non-homologous recombination, which may lead to cell transformation by insertional mutagenesis [31]. Conversely, mRNA molecule-based vaccines act in the cytoplasm and thus do not pose a risk of chromosomal integration.

#### DNA ZIKV vaccines

Inovio Pharmaceuticals and GeneOne Life Science, Inc. (KSE: 011000) have developed a synthetic, consensus DNA vaccine (GLS-5700) encoding the ZIKV premembrane (prM) and envelope (E) proteins, administered with the CELLECTRA^®^-3P device, Inovio’s proprietary intradermal DNA delivery device. The delivery technology is based on electroporation. The interim analysis of the phase 1, open-label clinical trial at 14 weeks (i.e., after the third dose of vaccine given in a 0–4 and 14 weeks schedule) evaluated the safety and immunogenicity of GLS-5700 in two groups of 20 participants each (NCT02809443) [32]. No serious adverse events were reported. After the third vaccine dose, binding antibodies (as measured on enzyme-linked immunosorbent assay) were detected in all participants. Neutralizing antibodies developed in 62% of the vaccine recipients on the Vero-cell assay. On a neuronal-cell assay, there was 90% inhibition of ZIKV infection in the serum samples of 70% of vaccine recipients and 50% inhibition in 95% of vaccine recipients. Further, the intraperitoneal injection of post-vaccination serum protected 103 of 112 (92%) IFNAR knockout mice that were challenged with a lethal dose of ZIKV-PR209 strain.

The US NIH Vaccine Research Center is advancing a ZIKV DNA vaccine candidate based on the technology it developed for a highly immunogenic West Nile virus DNA vaccine [33], whereby the full coding sequences of the prM and E genes of ZIKV are inserted into their DNA construct. In this manner, virus-like subviral particles are released after expression of prM and E [13]. The National Institute of Allergy and Infectious Diseases (NIAID) is using a needleless pressure-based delivery system developed by the company PharmaJet, with results from immunogenicity and protective efficacy studies in mice and in rhesus monkeys indicating high levels of protection [13]. The phase 1 clinical trial of this DNA vaccine started in September 2016 and a phase 2a clinical trial in Texas and Puerto Rico was initiated in April 2017 [34]. A phase 2b trial is scheduled to begin before the end of 2017 in multiple sites with the potential for ZIKV transmission [35].

#### mRNA vaccines

Modified ZIKV prM-E mRNA molecules were encapsulated in lipid nanoparticles in vaccine formulations [36, 37], showing complete protection in animal studies against challenge after a single intradermal immunization [38] or after prime and boost intramuscular immunization [39]. The nucleoside-modified mRNA ZIKV vaccine (mRNA-1325), which is being developed by Moderna, a Cambridge-based Biotech Company [36], entered a phase 1 clinical trial in December 2016 (NCT03014089). The mRNA candidate developed by NIAID and GlaxoSmithKline could enter clinical trials in late 2017.

#### Purified, inactivated whole virus vaccines (PIV)

The inactivation process eliminates virus replication while maintaining the antigenicity of the structural proteins, and thus PIV are thought to be safe during pregnancy. PIV vaccines have been successfully licensed for both JEV and TBEV. ZIKV PIV vaccines would most likely be less costly than nucleic acid vaccines. However, it is plausible that PIVs could require multiple doses in the primary schedule, adjuvants to enhance immunogenicity, and boosters to sustain protective immunity. ZIKV PIV derived from the Puerto Rico strain PRV ABC59 or from the MR 766 strain, produced in Vero cells, and inactivated with formalin, were tested in either Balb/c mice, rhesus monkeys, AG 129 mice, or New Zealand white rabbits and showed good induction of ZIKV-specific neutralizing antibodies [15, 16, 40]. Further, a ZIKV PIV candidate with an alum adjuvant is being evaluated in several phase 1 trials (NCT03008122, NCT02952833, NCT02963909, NCT02937233). The results of three phase 1 placebo-controlled, double-blind trials in healthy adults of ZIKV PIV with aluminum hydroxide adjuvant were recently published [41], showing only mild to moderate adverse events. By day 57, 92% of vaccine recipients had seroconverted (microneutralization titer ≥ 1:10), with peak geometric mean titres seen at day 43 and exceeding protective thresholds seen in animal studies. NIAID’s Vaccine Research Center will test a ZIKV PIV as a boost to its DNA Zika vaccine candidate. Bharat and Takeda are also developing a PIV against ZIKV.

#### Viral-vectored vaccine candidates

Viral-vectored vaccines share the same ease of production and stability with DNA plasmid vaccines and may therefore be easily scalable in epidemic situations. Viral-vectored vaccines induce both innate and adaptive immune responses in mammalian hosts [42]. Adenoviral vectors have been used to deliver ZIKV prM-E [40], and were shown to have higher neutralizing antibody titers and T-cell immunity than PIV, DNA, and protein subunit vaccines [15]. Nevertheless, limitations for adenovirus vaccines include their ability to induce toxic inflammatory responses and the potential for pre-existing immunity to naturally occurring human adenoviruses resulting in accelerated clearance and dampened immunogenicity [42]. Reactogenicity has been circumvented by the deletion of genes required for replication, which also allows for larger inserts [42]. Non-human primate adenoviruses as vaccine vectors can bypass pre-existing immunity to human adenoviruses. Adenovirus-vectored and chimpanzee adenovirus-vectored vaccines for ZIKV are still in pre-clinical development.

The core technology of the measles vector platform developed at the Institut Pasteur in Paris and now licensed to Themis Bioscience was successfully tested in a phase 1 trial for chikungunya virus [43]. The live recombinant measles virus-based chikungunya vaccine had good immunogenicity, even in the presence of anti-vector immunity, was safe, and had a generally acceptable tolerability profile, making this the first promising measles virus-based candidate vaccine for use in humans. With regards to ZIKV, the measles vaccine-ZIKV chimeric virus recently entered a phase 1 clinical trial (NCT02996890).

#### Subunit protein/virus-like particles (VLPs)

Subunit protein vaccines are attractive as a platform due to their potential for safe use in all populations, including pregnant women, depending on adjuvants. Subunit protein vaccines are produced by transfecting a plasmid encoding a gene sequence of interest into bacteria, yeast, or insect cells and utilizing the machinery within those cells to produce the protein from the gene sequence. Similar to the PIV approach, a disadvantage to subunit protein vaccines is that they are generally less immunogenic than live vaccines and therefore require multiple doses and adjuvants to achieve protective immunity. The advantage of VLPs is that the antigens are presented in their native conformation without the need for a replicating virus. Subunit protein and VLP ZIKV vaccines have not yet entered clinical evaluation.

#### Live attenuated vaccines including recombinant heterologous flavivirus-vectored vaccines

Live attenuated vaccines are usually a favored vaccine technology because of their ability to induce durable and effective adaptive immunity at relatively low production costs. Live vaccines mimic natural viral infections and thus induce a strong antibody and cell-mediated immunity. However, live attenuated vaccines induce transient low-grade viremia. As CZS is thought to occur even in asymptomatically infected pregnant women with low grade viremia [27], replicating live vaccines need to be carefully evaluated for their safety prior to their administration to women of reproductive age, some of whom may be inadvertently pregnant. However, similar to the approach to congenital rubella syndrome [44, 45], live attenuated Zika vaccines may play a significant role in endemic transmission use, for example, by their incorporation to childhood vaccination programs in countries with ZIKV transmission. As ZIKV is a neurotropic virus, neurovirulence and reproductive toxicology testing are critical early steps in the development of live attenuated vaccines prior to human studies. Demonstration of mosquito non-competence is also required.

Live attenuated replication-competent vaccines are available for recombinant (or chimeric) flaviviruses. The principle of chimerization is to insert target antigens (for example, prM and E) into a back-bone vector. Sanofi-Pasteur developed a recombinant ZIKV vaccine based on the yellow fever virus 17D backbone, which has been used to develop and license live attenuated recombinant DENV and JEV vaccines [46]. NIH/NIAID is also using recombinant DNA technology to design recombinant ZIKV/DENV viruses, a strategy employed in the creation of the DENV-2 component of TV003, rDEN2/4Δ30 [47]. For the ZIKV candidate vaccine, the prM and E coding sequences of ZIKV are being evaluated, replacing those of DENV-2 or DENV-4. Combining the NIH tetravalent DENV vaccine with the recombinant ZIKV/DENV component may provide a combination DENV-ZIKV vaccine, which could be useful for populations living in regions endemic for both.

### WHO’s target product profile for a ZIKV vaccine

Non-replicating platforms with no documented safety concerns for use during pregnancy would be the preferred vaccine platform for a ZIKV vaccine for emergency use where women of reproductive age are the primary target, ideally with a single dose primary series [6]. Vaccines based on replication-competent platforms are likely to have profiles more suitable for routine/endemic transmission use. As there is a theoretical risk that live, attenuated, or replication-competent viral vaccines given to pregnant women may be capable of crossing the placenta and infecting the fetus [48], live vaccines are generally not recommended for use during pregnancy. However, live attenuated vaccines have been given to women of child-bearing age (MMR, yellow fever, polio) in situations of increased risk of exposure, and inadvertent vaccination of pregnant women does occur in mass vaccination campaigns. To date, there is no evidence of increased adverse pregnancy outcomes due to immunization with a live attenuated vaccine [49]. However, the safety assessment and regulatory requirements for live attenuated/replicating-competent ZIKV vaccines are likely to require additional data compared to non-replicating vaccine platforms. Non-replicating vaccine platforms that either do not use any adjuvant or use a well-characterized adjuvant in currently licensed vaccines, such as aluminum salts (e.g., alum), would be preferable. However, the use of other adjuvants may be justifiable if accompanied with superior performance and delivery aspects (e.g., reduced number of doses).

## Zika therapeutics

Therapeutics against ZIKV need to be developed in parallel to vaccines and may have a specific role in reducing the burden of Zika infection and disease in the populations most at risk of serious outcomes. Drugs could rationally be used for prophylaxis or post-exposure prophylaxis to prevent or mitigate the severity of CZS, and may have particular value when low endemicity does not justify widespread immunization. Aborting ongoing ZIKV shedding in seminal fluids may be another indication. Antivirals are the cornerstone of management of chronic human viral infections like HIV, hepatitis B, and hepatitis C. There are also precedents for therapies to manage viral infection in pregnant women and their fetus such as post-exposure prophylaxis with immune immunoglobulins in susceptible women to protect the mother and fetus from infection with varicella. Any new drugs for ZIKV would be used as an adjunct to the standard of care for non-pregnant and pregnant persons, and may be indicated before vaccines become widely available or in addition to vaccine programs.

Three plausible clinical indications for application of a medicinal prophylactic/therapeutic against ZIKV are (1) to offer prophylaxis or early post-exposure prophylaxis, (2) to accelerate viral clearance, and (3) to reduce disease severity (Box 1).

### Human immune globulin and anti-ZIKV monoclonal antibodies (mAb) for prophylaxis or treatment

Human immune globulins are used clinically against some viral infections in pregnant women. For measles, the primary purpose is to attenuate disease in the pregnant woman and prevent perinatal transmission to the newborn. For varicella, the purpose is to prevent or attenuate disease in the pregnant woman and prevent congenital infection [50]. However, the incubation time of varicella is 2–3 weeks, far longer than for ZIKV (3–10 days), and therefore the critical time to treat is shorter for ZIKV. Plausibly, human immune globulin (or hyperimmune globulin) from ZIKV-immune donors, or human mAbs, could be used for prophylaxis or therapy. mAbs are promising because they can be precisely defined and their production controlled and scaled up. Blood from a ZIKV-immune donor and a human B-cell immortalization technique was used to identify human mAbs that bound ZIKV antigens (NS1 and E proteins) [51]. An EDIII-specific antibody, ZKA190, protected mice from lethal ZIKV infection, illustrating the potential for antibody-based therapy. Another mAb, ZIKV-117, was identified as broadly neutralizing of ZIKV infection in vitro [52]. Epitope mapping studies have revealed that ZIKV-117 recognized a unique quaternary epitope on the E protein dimer-dimer interface. Treatment of Zika-infected pregnant and non-pregnant mice with ZIKV-117 markedly reduced tissue pathology, placental and fetal infection, and mortality. A bispecific mAb has also been developed that could address concerns about the emergence of anti-viral resistance to monospecific mAbs [53]. Collectively, these data demonstrate the feasibility of developing mAbs as therapeutic and/or prophylactic candidates.

### Small molecule antivirals for prophylaxis or treatment

Multiple studies have demonstrated the anti-ZIKV activity of several Food and Drug Administration (FDA)-approved drugs or drug candidates being clinically tested for other indications [54–59]. For example, the anti-HCV prodrug Sofosbuvir has anti-Zika virus activity in vitro [54]; however, repurposing this compound is problematic because its hydrolysis is highly specific to the liver. Niclosamide, a category B anthelmintic drug, inhibited ZIKV replication at low micromolar concentrations [58]. However, the poor systemic bioavailability of niclosamide is a hurdle to further clinical development against Zika. More than 20 out of 774 FDA-approved drugs decreased ZIKV infection in an in vitro screening assay [54]. Selected compounds were further validated for inhibition of ZIKV infection in human cervical, placental, and neural stem cell lines, as well as in primary human amnion cells. Established anti-flaviviral drugs (e.g., bortezomib and mycophenolic acid) and others with no previously known antiviral activity (e.g., daptomycin) were identified as inhibitors of ZIKV infection. These results offer the possibility of a repurposed drug being used for Zika therapeutic or prophylactic indications.

Newly discovered candidate anti-virals include a synthetic peptide derived from the stem region of the ZIKV envelope protein, designated Z2, which potently inhibits infection of ZIKV and other flaviviruses in vitro [60]. Z2 is able to penetrate the placental barrier to enter fetal tissues and prevent vertical transmission of ZIKV in pregnant C57BL/6 mice [60]. Another molecule, galidesivir, is an adenosine analogue active in cell culture against a wide-range of RNA viruses [61]. Galidesivir treatment of ZIKV-infected mice significantly improved survival even when treatment was initiated 5 days after infection [62]. However, potential hurdles for galidesivir development is the requirement for an oral formulation (galidesivir requires parenteral administration). Ribavirin, another broad-spectrum but teratogenic antiviral, did not improve outcomes from ZIKV infection in the same model (Cristina Cassetti; personal communication). A summary of compounds found to have Zika antiviral properties in vitro (Table 3) and of some of the repurposed drugs reported to have anti-Zika activities are provided herein (Table 4).

**Table 3:** List of potential compounds for repurposing with anti-Zika activity, extracted from [19, 83]

Drug group	Drug name	Description
Nucleoside analogs	Sofosbuvir, MK-608	• Inhibit Zika virus (ZIKV) replication in cellular assays • Efficacious in animal models
	2CMC, Ribavirin, Favipiravir, T1105	• Showed antiviral activity in cell culture
	BCX4430, GS5734	• Reduced mortality in ZIKV-infected mice • Currently in phase I and II clinical trials
Peptidomimetic agents	CN-716	• Inhibit ZIKV protease in vitro, but only weakly inhibit viral replication • Due to safety reasons, may not translate as therapeutic option for pregnant women, but could be applied to other infected individuals
Adenosine analog	NITD008	• Showed potent anti-ZIKV activity • Could serve as a reference inhibitor for future drug screen and discovery
Cyclin-dependent kinase inhibitor	PHA-690509	• Showed inhibition of ZIKV replication of all three strains
Antimalaria	Chloroquine	• Reduces virus production, the number of infected cells, and cell death promoted by ZIKV infection without any cytotoxic effect • Promising candidate for ZIKV clinical trials • Can be safely administered to pregnant women since it is clinically approved
Anthelmintic	Bithionol	• Propagate by activating host caspases and inducing programmed cell death
Epigallocatechin gallate		• Natural compound found in food items, particularly green tea • It inhibits ZIKV entry into host cell
Interferon-inducible transmembrane proteins		• Inhibit the replication of a number of pathogenic viruses

**Table 4:** High throughput screening for potential compounds with anti-Zika activity (drug repurposing)

Study	No. of drugs screened	Compounds identified with anti-Zika activity	Remarks
Barrows et al. [54]	774 FDA-approved agents	Clofazimine, Digoxin, Gemcitabine, Ivermectin, Mefloquine, Mercaptopurine hydrate, Mycophenolic acid, Fingolimod, Mycophenolate mofetil, Dactinomycin, Bortezomib, Methoxsalen (Xanthotoxin), Azathioprine, Thioguanine, Auranofin, Sertraline, Pyrimethamine, Daptomycin, Palonosetron, Deferasirox, Micafungin, Sorafenib tosylate, Cyclosporine A, Mebendazole	• Mycophenolic acid (MPA), Ivermectin, Daptomycin, Mefloquine, Palonosetron identified as having higher potency • Daptomycin, Mefloquine, and Palonosetron are pregnancy category B drugs
Xu et al. [58]	6000 compounds, > 2000 FDA-approved agents	Niclosamide, Emricasan, 10 structurally unrelated inhibitors of CDK	• Emricasan is an inhibitor of caspase-3 activity but uncertain if a capase-3 inhibitor with anti-inflammatory properties impacts development of unborn fetus

## Challenges for clinical evaluation of Zika vaccines and therapeutics

Various challenges may delay or hinder the successful licensure of Zika vaccines or therapeutics, as described below.

### Selection of the most suitable clinical endpoint

In June 2017, WHO convened a meeting to elaborate on clinical endpoints for ZIKV vaccine efficacy trials [10]. Although preventing CZS is the outcome of greatest interest for public health, the large sample sizes required, the focus on women only, the heterogeneity of clinical manifestations of CZS, and ethical considerations render CZS as the primary endpoint unfeasible. A possible endpoint for clinical trials could be ZIKV infection (whether symptomatic or not), which would require a smaller sample size compared to a clinical endpoint. However, detecting asymptomatic ZIKV infections (as measured by seroconversion or sampling for virological detection) poses several challenges, including the requirement of very frequent blood, urine, and possibly semen collection so as not to miss the acute infection and achieve virological diagnosis [63]. Vaccination may also interfere with serological testing, e.g., it may render it difficult to discriminate between vaccine response and natural infection. A challenge with using clinical disease as the primary endpoint is that ZIKV illness is often associated with mild and non-specific symptoms, which raises challenges for case detection. A standardized clinical case definition is essential to facilitate the comparison and combining of information from different studies. A working case definition of virologically confirmed Zika illness has been provided by the Pan American Health Organization [64].

The consensus at the WHO technical consultation in June 2017 was to select virologically confirmed clinical illness as the primary endpoint, and to additionally study a subset to explore the protection against infection or reduction in viremia. The underlying assumption is that reduction in ZIKV disease incidence is associated with either sterilizing immunity or a reduction in ZIKV viremia, which in turn will reduce or prevent subsequent development of complications in pregnant and non-pregnant individuals.

### Inclusion of pregnant women in trial design and safety considerations

Although pregnant women would not be the primary target population for efficacy trials based upon the above rationale, pregnant women remain a priority population for ZIKV vaccine use in areas experiencing ongoing transmission and in future outbreaks. Thus, the Ethics Working Group on ZIKV Research and Pregnancy [65] recommended the collection of data specific to safety and immunogenicity in pregnancy for all ZIKV vaccine candidates to which pregnant women may be exposed and ensuring that pregnant women have fair access to participate in ZIKV vaccine trials that offer a favorable ratio of risks to potential benefits. Clinical development plans should therefore include systematic collection of relevant indicators and outcomes of safety and efficacy for pregnant women. Although certainly a complex challenge, a concerted and proactive effort is required to address the needs of pregnant women and their offspring early and across the ZIKV vaccine R&D pathway.

### Sample size and trial site selections

Generating clinical efficacy data in a reasonable sample size and an acceptable timeframe and cost is challenging at a time when global Zika incidence has declined to low levels. Areas with recent active ZIKV transmission may not be the best sites for clinical trials. Given that estimates of ZIKV seroprevalence are as high as 70% in some areas that experienced an outbreak, the proportion of susceptible individuals in such populations will be low, with a subsequent incidence too low to sustain an efficacy trial. Therefore, the WHO technical consultation in June 2017 proposed the projection of future evolution of the ZIKV epidemic based on the presence and vectorial capacity of *Aedes* mosquitoes [66, 67], travel patterns [68–70], and risk mapping and modeling [71–74] to predict the movement of Zika [75, 76]; various mathematical modeling groups are working to this end. A multi-site approach for vaccine trials will be needed to increase the chance of including populations with a high incidence of disease, as well as providing an opportunity to evaluate vaccine efficacy across different populations.

### Immune correlates

An immune correlate of protection is an immune response marker that is statistically associated with protection from disease or infection and may be either mechanistic (causally related to outcome) or non-mechanistic/surrogate (statistically related to outcome). Given the global decline in cases, it is unclear whether large scale efficacy trials are viable given the current incidence of ZIKV transmission. If clinical efficacy trials are not feasible, immune correlates/surrogates derived from passive protection studies in animals, natural history studies, and controlled human challenge study results may possibly represent acceptable endpoint data for initial emergency use authorization and eventual licensure. ‘Accelerated approval’ is based on the demonstration of a surrogate of protection though well-controlled clinical studies that are reasonably likely to predict clinical benefit. The US FDA ‘animal rule’ is based on the demonstration of an immune marker of protection in animal models that will reasonably likely predict clinical benefits in humans. Both accelerated approval and animal rule approaches require post-licensure studies to verify clinical benefit and safety. Controlled human infection models are a promising avenue to explore immune correlates in humans, however, they are associated with complex ethical considerations. The feasibility of establishing immune correlates or surrogates is now a priority.

### Assay optimization and standardization

A comprehensive review of ZIKV diagnostics was recently performed [77] and shortcomings highlighted [63]. In the context of a highly epidemic disease with an apparent short duration of detectable viremia and relatively infrequent incidence of clinical disease, reliable case ascertainment in efficacy trials is critical. However, the short and relatively low level viremia is difficult to detect, and the serological assays lack specificity because of cross-reactivity between other co-circulating flaviviruses and flavivirus vaccines [78]. Frequent sampling over time and sampling of various bodily fluids (whole blood, serum, urine), as well as the combination of various diagnostic assays will be necessary to increase the diagnostic yield. For the comparability of clinical trial results, it is crucial to standardize diagnostic assays used and immunological reference reagents should be available. The plaque reduction neutralization test is still considered to be the laboratory standard against which other neutralizing antibody assays should be compared. A guideline on plaque reduction neutralization test standardization can be found on the specific WHO website [79].

### Interaction between DENV and ZIKV

Given the widespread endemicity of DENV in the areas most affected by the current ZIKV outbreak, and the fact that short- or long-term immunological interaction between DENV and ZIKV cannot currently be excluded, trials would ideally need to take baseline blood samples for all subjects to ascertain prior DENV exposure in order to study the impact of prior immunity to DENV on vaccine performance and safety. For DENV vaccines, WHO recommends that subjects are followed-up for safety and efficacy for at least 3–5 years from the time of completion of primary vaccination due to the concern of immune enhancement [80]; however, given the lack of data supporting a clinical significant interaction between DENV and ZIKV [26–28], such a formal recommendation has not yet been made for Zika vaccine development. Nevertheless, a longer follow-up period to monitor safety could be considered.

### Establishing a transparent framework for selecting vaccines

Given the global decline in ZIKV incidence and the potential bottleneck in identifying suitable trial sites, a proposal was made during the June 2017 WHO technical consultation to establish a transparent framework for prioritizing vaccines to be evaluated in phase 2b/3 trials. Selection criteria would depend on the desired attributes, including compliance with the target product profile, pre-clinical evidence of complete or near-complete prevention or reduction of viremia, safety during pregnancy, and scalability of the product.

### Donor and industry fatigue

Major vaccine producers, government-funded institutions, academics, and small to mid-size research enterprises responded promptly to the Zika outbreak, setting aside other activities to focus on rapidly developing vaccines and therapeutics against Zika, supported by government and philanthropic funding agencies. However, with the rapid decline in cases, the unpredictability of future outbreaks, and the still poorly defined use scenarios, the commercial market has become questionable. The prospect of a licensed Zika vaccine is at stake unless governments and other donors sustain the level of support to advance development. Current models for stimulating epidemic product development are failing. The Coalition for Epidemic Preparedness Innovations (CEPI) is a new alliance between governments, industry, academia, philanthropy, intergovernmental institutions (such as WHO), and civil society, and was founded to finance and coordinate the development of new vaccines to prevent and contain infectious disease epidemics [81]. Zika is not yet on the priority list for CEPI, but as donor and industry fatigue may increase, CEPI, or such other mechanisms, will be needed to ensure that, out of the many Zika vaccine candidates, at least one will make it to the finish line.

## Conclusion

At least 45 Zika vaccine candidates have been or are in development, some of them already in phase 2 clinical trials. Multiple vaccine platforms have shown robust protection against ZIKV challenge in animal models. However, unique challenges will need to be addressed in the clinical development and regulatory pathways of a ZIKV vaccine that may hinder the development, licensure, and WHO-prequalification of high-quality, safe, and effective ZIKV vaccines. Implementing phase 3 efficacy trials will be difficult given the challenges of the spatial and temporal heterogeneity of ZIKV transmission, the unpredictability of the ZIKV epidemics, the broad spectrum of clinical manifestations making a single definite endpoint difficult, the lack of sensitive and specific diagnostic assays, and the need for inclusion of vulnerable target populations. In addition to a vaccine, drugs for primary prophylaxis, post-exposure prophylaxis, or treatment should also be developed in order to prevent or mitigate the severity of CZS. The global research and public health community should prioritize the development of ZIKV vaccines and therapeutics that will be acceptable for use by women of reproductive age, and ensure availability and affordability for use in countries where ZIKV is circulating. To this end, WHO is working towards a roadmap for Zika vaccine and product development.

## Box 1: Clinical indications for application of a prophylactic/therapeutic against Zika

***INDICATION 1. PROPHYLAXIS OR EARLY POST-EXPOSURE PROPHYLAXIS.***

***(1) To prevent maternal infection and fetal disease:***

The objective is to prevent, or diminish, Zika virus (ZIKV) infection and disease in pregnant women, or women trying to become pregnant, and thus eliminate or substantially reduce the probability of intrauterine infection or transmission in the perinatal or postnatal period.

- **Examples of this approach for Zika.** Prophylaxis for a pregnant woman living in a setting where there is epidemic Zika transmission, or a pregnant traveler spending time in a Zika-affected location. Post-exposure prophylaxis might be considered where there is strong suspicion that a pregnant woman has been exposed to Zika because of epidemiological circumstances (e.g., lives in an endemic area and resides in a household where recent Zika cases have been diagnosed)
- **Challenges.** The risk/benefit of using prophylactic agents (small molecule drugs or immune globulin) must be balanced against the probability of the mother and fetus being infected and of that infection harming the fetus
- **Examples of this approach for other infectious diseases.** Varicella virus (chickenpox) infection during pregnancy may result in congenital varicella, which is usually benign and self-limiting, but can occasionally produce a characteristically severe pattern of abnormalities known as ‘congenital varicella syndrome’. Zoster immunoglobulin (a preparation of human Ig containing anti-varicella antibodies) is indicated for all pregnant women who have significant exposure to Varicella Zoster virus infection (defined as ‘living in the same household as a person with active chickenpox or herpes zoster or face-to-face contact with a person with chickenpox or uncovered zoster for at least 5 minutes’), who have no history of chickenpox and who are seronegative.

***(2) To prevent Guillain–Barre Syndrome or other ZIKV infection-related neurological complications.***

Prophylaxis throughout the duration of exposure (e.g., travel to a Zika endemic area).

Early post-exposure prophylaxis after known exposure to a Zika case (e.g., sexual exposure, nosocomial exposure such as needle stick injury, living in same household of a current Zika case).

***INDICATION 2. THERAPY TO ACCELERATE RESOLUTION OF INFECTION***

- **Examples of this approach for Zika.** A pregnant woman, who lives in a setting where ZIKV is known to circulate, presents to a clinic with clinical signs and symptoms that could represent ZIKV infection. Out of an abundance of caution, empiric treatment commences before the results of laboratory tests are known (if such tests are available). The purpose of treatment is to accelerate clearance of virus infection from the maternal tissues and mitigate the likelihood of intrauterine or peri- or postnatal virus infection. A second example of treatment is for neonates who have acquired ZIKV from intrauterine exposure or perinatally.
- **Challenges.** As for prophylaxis, the use of a therapeutic agent must be balanced by the safety and cost profile of the drug and the likelihood that treatment will deliver clinical benefits to the fetus, i.e., prevent or modify intrauterine infection. For many Zika cases, viremia is already in rapid decline, or even undetectable by the time the patient presents to healthcare providers. An additional delay is created if treatment is guided by laboratory diagnostics rather than an empiric approach.
- **Examples of this approach for other infectious diseases.** There are no examples for acute viral infections, but in the setting of chronic infections such as HIV, it is well accepted that vertical transmission is RNA copy number-dependent, with higher rates occurring with increasing viral loads present in the mother. Treatment of pregnant women has been demonstrated to drastically reduce the incidence of vertical transmission in women undergoing treatment with a combination of antiretroviral compounds [82].

***INDICATION 3. DISEASE MODIFICATION FOR EXISTING CONGENITAL INFECTION.***

The objective is the treatment of existing intrauterine fetal infection by eradication of virus and thus reduce the severity of congenital Zika syndrome.

- **Examples of this approach for Zika.** A pregnant woman has amniocentesis performed because of concerns about recent exposure to ZIKV. The amniocentesis fluid is RT-PCR positive for ZIKV. Treatment is commenced to eradicate virus from the fetal tissues.
- **Challenges.** This indication is unlikely to be the sole basis for drug development. Clinical trials to test for improvements in fetal outcome would be very long in duration and likely prohibitively expensive to perform. The extent of viremia in the pregnant woman before commencement of treatment may already have led to significant pathology of the fetus.
- **Examples of this approach for other infectious diseases.** Unfortunately, there is no evidence that the outcome of an established congenital viral infection (e.g., with cytomegalovirus or varicella) can me modified by small molecule drug or immunoglobulin treatment.

## Abbreviations

CEPI: Coalition for Epidemic Preparedness Innovations
CZS: congenital Zika syndrome
DENV: Dengue virus
FDA: Food and Drug Administration
GBS: Guillain–Barre syndrome
JEV: Japanese encephalitis virus
mAb: monoclonal antibodies
mRNA: messenger RNA
NIH: National Institutes of Health
PIV: purified, inactivated whole virus vaccines
R&D: research and development
TBEV: tick-borne encephalitis virus
VLP: virus-like particles
WHO: World Health Organization
ZIKV: Zika virus
